# Nitric Oxide Synthase Neurons in the Preoptic Hypothalamus Are NREM and REM Sleep-Active and Lower Body Temperature

**DOI:** 10.3389/fnins.2021.709825

**Published:** 2021-10-14

**Authors:** Edward C. Harding, Wei Ba, Reesha Zahir, Xiao Yu, Raquel Yustos, Bryan Hsieh, Leda Lignos, Alexei L. Vyssotski, Florian T. Merkle, Timothy G. Constandinou, Nicholas P. Franks, William Wisden

**Affiliations:** ^1^Department of Life Sciences, Imperial College London, London, United Kingdom; ^2^Wellcome-MRC Institute of Metabolic Science and MRC Metabolic Diseases Unit, University of Cambridge, Cambridge, United Kingdom; ^3^Department of Electrical and Electronic Engineering, Imperial College London, London, United Kingdom; ^4^Institute of Neuroinformatics, University of Zürich/ETH Zürich, Zurich, Switzerland; ^5^United Kingdom Dementia Research Institute Care Research and Technology, Imperial College London, London, United Kingdom; ^6^Centre for Neurotechnology, Imperial College London, London, United Kingdom; ^7^United Kingdom Dementia Research Institute at Imperial College London, London, United Kingdom

**Keywords:** preoptic hypothalamus, nitric oxide, sleep, calcium photometry, body temperature, tetanus-toxin light-chain

## Abstract

When mice are exposed to external warmth, nitric oxide synthase (NOS1) neurons in the median and medial preoptic (MnPO/MPO) hypothalamus induce sleep and concomitant body cooling. However, how these neurons regulate baseline sleep and body temperature is unknown. Using calcium photometry, we show that NOS1 neurons in MnPO/MPO are predominantly NREM and REM active, especially at the boundary of wake to NREM transitions, and in the later parts of REM bouts, with lower activity during wakefulness. In addition to releasing nitric oxide, NOS1 neurons in MnPO/MPO can release GABA, glutamate and peptides. We expressed tetanus-toxin light-chain in MnPO/MPO NOS1 cells to reduce vesicular release of transmitters. This induced changes in sleep structure: over 24 h, mice had less NREM sleep in their dark (active) phase, and more NREM sleep in their light (sleep) phase. REM sleep episodes in the dark phase were longer, and there were fewer REM transitions between other vigilance states. REM sleep had less theta power. Mice with synaptically blocked MnPO/MPO NOS1 neurons were also warmer than control mice at the dark-light transition (ZT0), as well as during the dark phase siesta (ZT16-20), where there is usually a body temperature dip. Also, at this siesta point of cooled body temperature, mice usually have more NREM, but mice with synaptically blocked MnPO/MPO NOS1 cells showed reduced NREM sleep at this time. Overall, MnPO/MPO NOS1 neurons promote both NREM and REM sleep and contribute to chronically lowering body temperature, particularly at transitions where the mice normally enter NREM sleep.

## Introduction

Numerous circuits dispersed throughout the brain induce NREM sleep, but the preoptic (PO) hypothalamus, one of the first sleep-promoting centers to be identified (Nauta, 1946), has a major role (Sherin et al., 1996; Zhang et al., 2015; Weber and Dan, 2016; Chung et al., 2017; Kroeger et al., 2018; Ma et al., 2019; Reichert et al., 2019; Reitz and Kelz, 2021). The PO area also contains neurons that are required for REM sleep (Lu et al., 2000), including REM-promoting cells in MPO (Suntsova and Dergacheva, 2004; Gvilia et al., 2006). The PO area, which contains a huge diversity of cells (Moffitt et al., 2018; Tsuneoka and Funato, 2021), also contributes to regulating many other functions, including nesting, thermoregulation, parenting, sexual behavior, water consumption, blood osmolarity, and daily torpor (Nakamura and Morrison, 2008, 2010; Morrison and Nakamura, 2011; Saper and Lowell, 2014; Abbott and Saper, 2017; Hrvatin et al., 2020; Takahashi et al., 2020; Tsuneoka and Funato, 2021).

The medial (M) and median (Mn) PO hypothalamic areas are enriched for neuronal nitric oxide (*nos1*) gene expression, as seen by *in situ* hybridization in the Allen Brain Atlas (Lein et al., 2007), and from our previous studies (Harding et al., 2018). Previously we found that NOS1 neurons in the MnPO and MPO area link NREM onset and the decrease of body temperature that accompanies sleep (Harding et al., 2018). We hypothesized that external warm sensing and NREM sleep induction through these neurons may be part of an energy conservation mechanism that optimizes sleep toward thermoneutral temperatures (Harding et al., 2020).

In addition to presumably synthesizing NO in response to excitation and calcium, MnPO/MPO NOS1 cells, depending on subtype, likely release both GABA and glutamate and/or various peptides (Moffitt et al., 2018). Here we show by calcium photometry that NOS1 neurons in MnPO/MPO have their highest activity during NREM sleep, becoming particularly active at the boundary of wake to NREM transitions, and they are also active during the latter parts of REM sleep episodes. Synaptic silencing of MnPO/MPO NOS1 cells with tetanus toxin light-chain (TeLC) expression induced bidirectional changes to NREM sleep structure: over the 24-h cycle, mice had less NREM sleep in the dark phase, and more in the light phase. Dark phase REM sleep also consolidated to longer episodes, with a reduction in REM transitions; however, both light- and dark-phase REM sleep had more delta and less theta power than in controls, possibly suggesting disrupted REM function. In addition, a shift in the core body-temperature profile to warmer temperatures and a disrupted siesta (ZT16-20) period were observed. Thus, vesicular release of transmitters from MnPO/MPO hypothalamic NOS1 neurons is needed for maintaining normal sleep and temperature profiles.

## Materials and Methods

### Mice

Experiments were performed under the Animals (Scientific Procedures) Act (1986) and approved by the local ethics committee. The mice used were *Nos1-ires-Cre^TM 1(cr^****^*E)*^****^*Mgmj*^/J* (JAX labs stock 017526), referred to here as *Nos1-Cre* mice, donated by Martin G Myers (Leshan et al., 2012), and C57BL/6J mice (supplied by Charles River United Kingdom). All mice used in the experiments were male and congenic on a C57BL/6J background. Mice were maintained on a reversed 12 h:12 h light:dark cycle at constant temperature (22 ± 1°C) and humidity with *ad libitum* food and water.

### AAV Transgenes and AAV Production

We used the following *pAAV* transgene plasmids: *pAAV-FLEX-GFP-TeLC* (Murray et al., 2011), and *pAAV-FLEX-GFP* (Addgene #28304, a gift from Edward Boyden). Plasmid *pAAV-FLEX-GCaMP6s* was created by inserting the *GCaMP6s* open reading frame from *pCMV-GCaMP6s* (Addgene plasmid 40753, gift of Douglas Kim) (Chen et al., 2013), into the backbone of *pAAV-flex-hM3Dq-mCHERRY* (Krashes et al., 2011) in place of the *hM3Dq* sequence, but retaining the loxP sites. AAV transgenes were packaged in-house into capsids with a 1:1 ratio of AAV1 and AAV2 capsid proteins. The adenovirus helper plasmid *pFΔ6*, the AAV helper plasmids *pH21* (AAV1) and *pRVI* (AAV2), and the *pAAV* transgene plasmids were co-transfected into HEK293 cells and AAVs harvested on heparin columns, as described previously (Klugmann et al., 2005; Yu et al., 2015). AAVs titers were determined with an AAVpro Titration Kit (for real-time PCR) Ver. 2 (TakaRa Bio). The virus titers were as follows: *AAV-FLEX-GCaMP6s*, 1.6 × 10^6^ viral genomes/μl; *AAV-FLEX-GFP-TeLC* 5.1 × 10^5^ viral genomes/μl; *AAV-FLEX-GFP*, 6.1 × 10^6^ viral genomes/μl.

### Surgeries and Stereotaxic Injections of AAV

Mice underwent their first surgery at 10-weeks old. The mice required two rounds of surgery including implantation of an abdominal temperature logger, followed one week later by stereotaxic injections of AAV virus and electrode placement for electrocorticography (ECoG). For surgery, mice were anesthetized with 2% isoflurane and given appropriate analgesia. Viral infusions were performed using a steel injector (10 μl-Hamilton #701) and the aid of an electronic pump. Injections were optimized for the target with injection volumes of between 0.05 and 0.2 μl at 0.1 μl min^–1^. The injection coordinates relative to Bregma were AP +0.34 mm, ML 0 mm, DV −4.8 and 5.2. A minimum of one week recovery was allowed before recording the EEG.

### EEG and EMG Recordings, Scoring of Vigilance States and Power Spectrum Analysis

EEG and EMG were recorded from non-tethered animals using Neurologger 2A devices as described previously and electrodes placed at the same positions as our previous work in mice (Anisimov et al., 2014; Zhang et al., 2015). These positions were: AP +1.5 mm, ML −1.5 mm relative to Bregma, 1st − AP −1.5 mm, ML +1.5 relative to Bregma, 2nd Lambda −1.0 mm, ML 0.0 mm. EMG wires were also implanted in the neck muscles. Data were recorded at a sampling rate of 200 Hz with four times oversampling. The EEG data analyzed using Spike2 software 7.18 (Cambridge Electronic Design, Cambridge, United Kingdom) or MATLAB (MathWorks, Cambridge, United Kingdom). Prior to sleep scoring the ECoG was digitally filtered (high-pass, 0.5 Hz, −3dB) and the EMG was band-pass filtered (5–45 Hz, −3dB). Power in the delta (1–4 Hz) and theta (6–9 Hz) bands was calculated, together with the RMS value of the EMG signal (averaged over 5 s), and these were used to define the vigilance states of wake, NREM and REM with an automatic script OSD7 v7.2 (in Spike2). Each vigilance state was then rechecked manually. We analyzed the sleep-state specific power spectrums following normalization to wake power within each mouse, as described previously (Ma et al., 2019).

### Photometry Recordings

Photometry was performed using a 473-nm diode-pumped solid state (DPSS) laser with fiber coupler (Shanghai Laser and Optics century Co.) and adjustable power supply (Shanghai Laser and Optics century Co.), controlled by a Grass SD9 stimulator. A lock-in amplifier (SR810, Stanford Research Systems, CA, United States) drove the laser using a TTL signal at 125 Hz with an average power of 80 μW at the tip of the fiber. Using an optical fiber patch cord (Ø 200 μm, 0.22 NA, Doric Lenses) the light source passed through a fluorescence cube (FMC_GFP_FC, Doric Lenses) and then *via* a second optical patch cord (Ø 200 μm, 0.37 NA, Doric Lenses), was connected to the brain-implanted fiber *via* a ceramic sleeves (Thorlabs). The GCaMP6s output was then filtered at 500–550 nm (using a fluorescence cube) and passed to a photodiode (APD-FC, Doric Lenses) and amplified by the lock-in amplifier (time constant, 30 ms). The signal was recorded on a CED 1401 Micro box (Cambridge Electronic Design, Cambridge, United Kingdom) at 200 Hz using Spike2 software (Cambridge Electronic Design, Cambridge, United Kingdom). The maximum continuous recording length was 6 h. Photometry, EEG and EMG data were aligned offline using Spike2 and analyzed using this software or custom scripts in either MATLAB (MathWorks) or R scripts (R Core Team, 2020). Peak counting was performed using Spike2 (using peak mode), “Peaks” were counted when immediately followed by a decrease of at least the threshold amplitude (100 μV) and were outside the minimal interval between detections of 10 ms. For each transition, the photometry signal *F* was normalized to baseline using the function Δ*F/F* = *(F−F_0_)/F_0_*, where *F*_0_ is the baseline fluorescence prior to the transition. Data are presented as a percentage. Heatmaps are shown as *Z*-scores. Transitions coinciding with recording artifacts or large shifts in baseline (DC offset) were excluded.

### Temperature Recordings

Core body temperatures were measured using an abdominally implanted temperature loggers (DSTnano, Star-Oddi, Herfølge, Denmark), sampling every 2 min, as described previously (Harding et al., 2018).

### Immunohistochemistry

Mice were given pentobarbital (100 mg/kg body weight; *i.p.*), and transcardially perfused with 4% paraformaldehyde in phosphate-buffered saline (PBS), pH 7.4. Brains were removed and 40-μm-thick coronal sections cut using a Leica SM 2010R microtome. Staining was performed on free-floating sections, washed in PBS three times and permeabilized in PBS plus 0.4% Triton X-100 for 30 min, blocked by incubation in PBS plus 10% normal goat serum (NGS), 0.2% Triton X-100 for 1 h (all at room temperature) and subsequently incubated overnight with a 1:1000 dilution of anti-GFP polyclonal antibody (A-6455, ThermoFisher). Sections were washed three times in PBS before incubating with goat anti-Rabbit IgG (H + L) Secondary, Alexa Fluor^®^ 488 conjugate (A-11034, ThermoFisher) for 2 h. Samples were then washed six times before mounting on Vectashield Antifade Mounting Medium with DAPI (H-1200, Vector Laboratories).

### Statistics

Data collection were either randomized or performed in a counter-balanced manner. Data are represented as the mean ± SEM, unless otherwise stated. OriginPro 2017 was used for statistical analyses. For data that were not independent (where ANOVA was not appropriate) we employed either two-tailed or paired *t*-tests and then accounted for multiple comparisons using the Benjamini-Hochberg procedure at a false discovery rate of 5%. Mice were excluded from the analysis if the histology did not confirm AAV transgene expression in the MnPO/MPO area, or if the expression had spread beyond the target region. Investigators were not blinded to behavioral treatment groups.

## Results

### Medial Preoptic Nitric Oxide Synthase 1 Neurons Are Most Active During NREM Sleep

**FIGURE 1:**
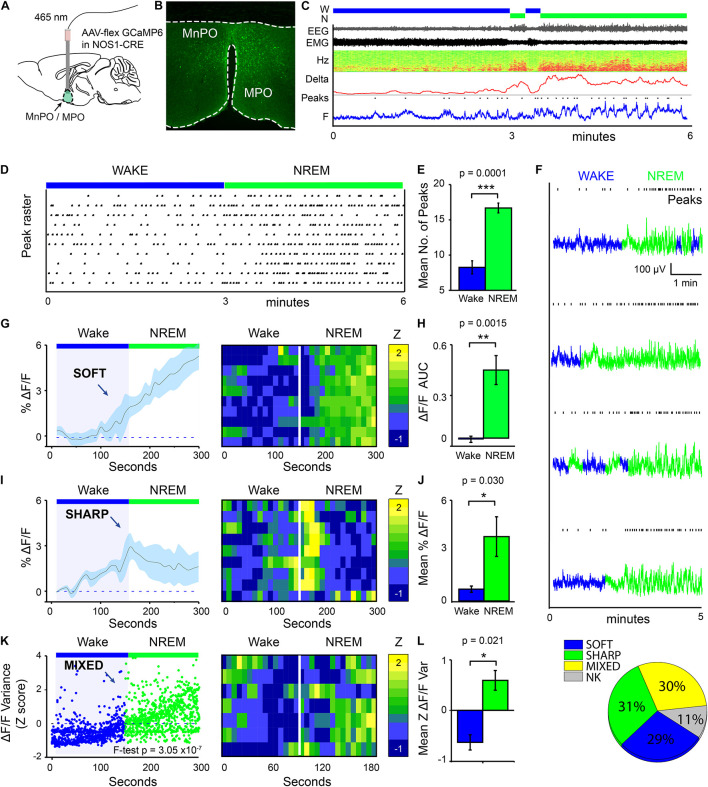
MnPO/MPO hypothalamic NOS1 neurons are more active during sleep. Animals were recorded for 6 h across the light cycle from lights-off to lights-on to facilitate a distribution of sleep states. Transitions are shown over 300 s. **(A)** Schematic for the photometry recording at a 5-mm depth and an example of the GCaMP6 expression site in a *Nos1-MnPO/MPO-GCaMP6s* mouse. **(B)** Expression of GCaMP6s in neurons in the MnPO/MPO hypothalamus as detected by immunocytochemistry with GFP antisera. **(C)** Example transition from wake to NREM over a 6-min interval. Also shown are scored sleep states (Wake, W; NREM, N; REM, R), Filtered EEG and EMG, spectrogram of power in the frequency domain over time (Hz), Delta power (1–4 Hz) with a 5-s root mean square (RMS), raw photometry signal (labeled F) and automated peak counting on the photometry signal (Peaks). **(D)** A raster plot of automated spike counting from calcium photometry signals across wake to NREM transitions. **(E)** Area under the curve (ΔF/F) between wake and NREM for soft transitions in calcium signal (Paired *t*-test, *n* = 5, *p* = 0.0001). **(F)** Raw photometry data with paired automated peak counting for four example transitions over 5 min, colored by sleep state. Wake is shown in blue and NREM is shown in green. Peaks in calcium are marked above each trace. **(G)** The average ΔF/F in calcium signal for soft-type transitions that increase across wake-NREM transitions and ten example transitions represented as a heatmap. **(H)** ΔF/F Area under the curve between wake and NREM for soft transitions (Paired *t*-test, *n* = 5, *p* = 0.002). **(I)** The average ΔF/F for sharp-type increases in calcium signal in wake-NREM transitions before returning to baseline, followed by ten example transitions represented as a heatmap. **(J)** Mean ΔF/F of the calcium signal between wake (baseline) and NREM (peak) for sharp transitions (Paired *t*-test, *n* = 5, *p* = 0.004). **(K)** The *z*-score of ΔF/F variance for mixed-type transitions in calcium signal that increase across wake-NREM transitions (*F*-test of all pooled transitions, *p* = 3 × 10^–7^). Shown alongside ten example transitions in the calcium signal represented as a heatmap. **(L)** Quantification of variance in the ΔF/F calcium signal in across mice (Paired *t*-test, *n* = 5, *p* = 0.021). **(M)** Proportion of each transition in calcium signal type (soft increase, sharp increase, mixed increase) found in all wake to NREM transitions. Transitions in calcium signal that could not be classified are labeled NK. The Benjamini-Hochberg procedure was used to account for multiple comparisons at a false discovery rate of 5%. *P < 0.05, **P < 0.01, ***P < 0.001.

We used calcium photometry to assess the sleep-wake activity of NOS1 neurons in the MPO area. *AAV-FLEX-GCaMP6s* was injected into the MnPO/MPO area of *Nos1-Cre* mice to generate *Nos1-MnPO/MPO-GCaMP6s* mice (Figures 1A,B). We then recorded calcium photometry signals from mice over 6 h while the mice behaved freely in their home cages. Many NOS1 neurons in the MnPO/MPO region were NREM sleep-active, having their highest calcium activity in NREM sleep with only sporadic activity during wakefulness. An example over a 6-min period of a transition to NREM sleep is shown in Figure 1C, alongside the raw photometry signal, delta power (1–4 Hz), spectrogram from 0 to 20 Hz, EEG, EMG and scored sleep state. During wakefulness only low-level calcium-induced fluorescence signal was seen (labeled “F” on the axis of Figure 1C), and peaks in the signal were rare. While occasional small peaks in the calcium signal occurred during wake, a specific increase in peak frequency in the calcium signal was associated with NREM sleep. This is shown as a raster plot for ten transitions in Figure 1D over a 6-min period and quantified in Figure 1E. Higher GCaMP6s signal levels and more frequent peaks occurred during NREM sleep. Four example photometry traces are shown in Figure 1F and color coded by sleep state. Peak counting is shown above each example.

On transitioning to NREM sleep the overall calcium levels in MnPO/MPO NOS1 neurons increased, as did the frequency of peaks in calcium signal. To quantify these changes in calcium signals, we averaged across multiple wake-NREM transitions from multiple mice. These averages contained several profiles: a slower “soft” rising transition in calcium signal with more peaks; and a faster “sharp” profile. Soft transitions from wake to NREM sleep are shown in Figure 1G, plotted as ΔF/F and averaged across 5 min of recording, alongside a heat map of ten example transitions. The ΔF/F calcium signal started to rise from the point of transition, and this continued for at least 150 s. This is quantified as the area under the curve in Figure 1H. On the other hand, sharp transitions in calcium-induced signals in MnPO/MPO NOS1 were different and anticipated the next NREM sleep transition. These sharp transitions are shown in Figure 1I as ΔF/F, alongside a heatmap of ten example transitions. Here, the ΔF/F signal started to rise up to 60 s prior to the start transition and peaked within 30 s of entering NREM sleep, before reducing again by 1 min post-transition. The heatmap shows that these calcium events were time-locked to the wake-NREM transitions. Within 100 s, the calcium signal had almost returned to baseline, despite continuous NREM. This was quantified as the mean amplitude between baseline and the maximum value in Figure 1J. Both the soft and sharp transitions in calcium signals had a slow time course that took more than 60 s to complete. The remainder of the calcium signals associated with the transitions from wake to NREM sleep of MnPO/MPO NOS1 neurons could not be classified into soft or sharp profiles. However, when these remaining calcium signals at the wake to NREM transitions were pooled and analyzed by variance, a clear association with the wake to NREM transitions was seen. These “mixed” transitions in calcium signals are shown in Figure 1K, plotted as a *Z* score of the ΔF/F variance, alongside a heatmap of ten example transitions. Here, the variance in calcium signal increased across the wake to NREM transitions in four of the five animals measured (Figure 1L). Overall, on moving from wake to NREM sleep, approximately 60% of changes in the calcium signal of MnPO/MPO NOS1 were either soft or sharp increases in signal, and 30% were mixed. Approximately 10% of the recordings of calcium signals did not show changes in activity during wake to NREM transitions (Figure 1M).

**FIGURE 2:**
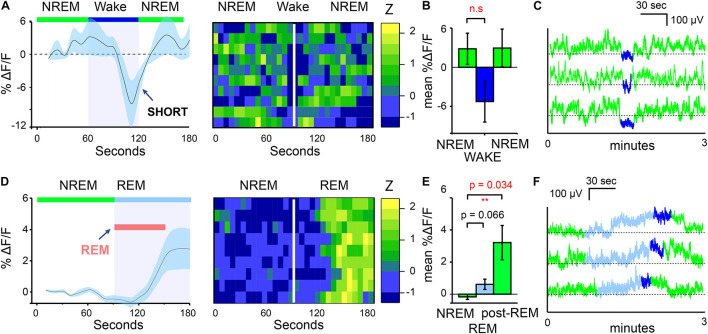
MnPO/MPO hypothalamic NOS1 neurons are active during the later part of REM episodes. Animals were recorded for 6 h across the light cycle from lights-off to lights-on to facilitate obtaining a distribution of sleep states. Transitions are shown over 180 s. **(A)** The average ΔF/F in calcium signal when NREM is interrupted by short bouts of wake. Wake length here is approximated and followed by ten example transitions, represented as a heatmap. **(B)** Mean change in ΔF/F calcium signal between brief wakefulness, prior-NREM and post-NREM for short transitions (Paired *t*-test, *n* = 4, prior-NREM vs. brief wakefulness, *p* = 0.063, post-NREM vs. brief wakefulness *p* = 0.076). **(C)** Examples of NREM to wake transitions from raw photometry data colored by sleep-state (NREM, green; REM, cyan and wake, blue). **(D)** The average ΔF/F for NREM to REM transitions showing no clear changes in signal with a slower increase after approximately 30 s alongside a heat map of nine transitions from NREM to REM sleep. **(E)** Mean change in ΔF/F calcium signal between the end of a NREM period and the later part of a REM episode (Paired *t*-test, *n* = 5, *p* = 0.066) and NREM and post-REM (Paired *t*-test, *n* = 5, *p* = 0.034). **(F)** Examples of calcium signals during NREM to REM to microwake to NREM transitions, colored by sleep-state (NREM, green; REM, cyan; wake, blue). **P < 0.01, n.s, not significant.

In addition to the increases in calcium signals of MnPO/MPO NOS1 neurons on transition from wake to NREM, we also looked at their calcium signals during transitions from NREM to wake and from NREM to REM sleep (Figure 2). We found that NREM sleep was sometimes interrupted with short bouts of wake episodes lasting about one minute (micro-wakes). In these short transitions, a small decrease in the calcium signal of MnPO/MPO NOS1 neurons was seen on entry into wakefulness that continued to decline until the next NREM episode and the calcium signal increased once more (Figures 2A,B). In this case, while we noted some clear examples, as shown in Figure 2C, there were also large variations between animals and there was insufficient statistical power to infer if these small decreases in calcium signal were significant (Figure 2B). The calcium signals in MnPO/MPO NOS1 neurons during the transitions from NREM to REM are shown in Figure 2D and shown alongside a heatmap of nine example transitions. As described above, once NREM has commenced the calcium signal tends to decay to a lower baseline (Figure 1I). At the transitions of NREM to REM, the average ΔF/F calcium signal remained at a low baseline for at least 30 s into the REM bout (red bar in Figure 2D). Following this there was an increase in the signal and plateau that lasted approximately 60 s (Figures 2D,E), which continued into micro-wake bouts before reentry into NREM (see examples Figure 2F).

### Medial Preoptic Nitric Oxide Synthase 1 Neurons Influence Sleep-Wake Structure

**FIGURE 3:**
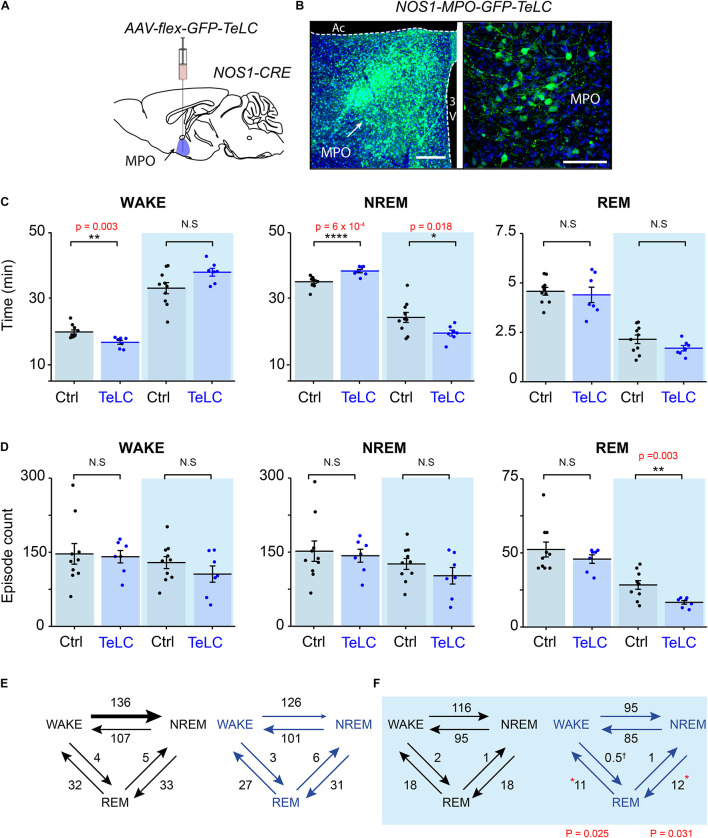
Reducing transmitter release from NOS1 neurons in MnPO/MPO hypothalamus alters sleep amounts and the number of sleep episodes in a manner dependent on the light-dark cycle. **(A)** Schematic of the stereotaxic injection of *AAV-flex-GFP-TeLC* into the MnPO/MPO area of *Nos1-CRE* mice to generate *Nos1-MnPO/MPO-GFP-TeLC* mice. **(B)** Example histology from MnPO/MPO showing expression of the GFP-TeLC protein as detected with a GFP antibody; left picture, lower magnification view, scale bar is 200 μm, right picture, higher magnification view, scale bar 100 μm. **(C)** Quantification of sleep states for each mouse in the 12-h light or dark periods shown as average time in vigilance state per hour. Wakefulness in the light phase (two-tailed *t*-test, *n* = 7 and *n* = 10, *p* = 0.003), NREM in the light phase (two-tailed *t*-test, *n* = 7 and *n* = 10, *p* = 0.0006), NREM in the dark phase (two-tailed *t*-test, *n* = 7 and *n* = 10, *p* = 0.018). **(D)** The number of episodes of wake, NREM and REM between light and dark. REM in the dark phase (two-tailed *t*-test, *n* = 7 and *n* = 10, *p* = 0.003). **(E)** Analysis of sleep transitions between sleep states in the light phase. No differences were observed between groups. **(F)** The transitions between *Nos1-MnPO/MPO-GFP-TeLC* mice and *Nos1-MnPO/MPO-GFP* mice in the dark phase. Transitions from NREM to REM (*p* = 0.025) and from REM to wake (*p* = 0.031), from two-tailed *t*-test, *n* = 7 and *n* = 10. Multiple comparisons were accounted for using the Benjamini-Hochberg procedure at a false discovery rate of 5%. *P < 0.05, **P < 0.01, N.S, not significant.

Having established that many NOS1 neurons in the MnPO/MPO area are more active during NREM and REM sleep than they are during wake, we next examined their contribution to sleep structure. To do this we reduced synaptic transmission from these cells, using Cre-dependent expression of tetanus-toxin light-chain (GFP-TeLC) (Figure 3A). Tetanus-toxin light chain blocks release of neurotransmitter vesicles by cleaving synaptobrevin, a synaptic vesicle protein (Schiavo et al., 1992). *AAV-FLEX-GFP-TeLC* or *AAV-FLEX-GFP* were injected into the MnPO/MPO of *Nos1-Cre* mice to generate *Nos1-MnPO/MPO-GFP-TeLC* and control *Nos1-MnPO/MPO-GFP* mice, respectively (Figure 3B). Reducing synaptic transmission from NOS1 neurons produced small but significant alterations to the structure of sleep (Figure 3C). These data are quantified for each mouse. Average wakefulness was reduced by almost 25% during the light phase, with a corresponding increase in NREM of approximately 10%. This was followed by an approx. 15–20% decrease in NREM during lights OFF. No clear changes were seen in REM sleep time.

We assessed whether changes in sleep structure seen in *Nos1-MnPO/MPO-GFP-TeLC* mice affected sleep episode dynamics and/or transitions (Figure 3D). There were no changes in the overall number of episodes in wake or NREM sleep for either the light or dark phase of the cycle; however, there was an approximately 45% reduction in REM episodes in the dark phase (Figure 3D). Consistent with this result, the number of NREM-REM and REM-wake transitions, but not transitions between wake and NREM sleep, were reduced by approximately 40%. No changes were seen in the light phase (Figures 3E,F). Although this was not reflected in the REM sleep amount, it was consistent with less NREM in the dark phase. In addition, we expected the remaining REM sleep to be consequently more consolidated.

**FIGURE 4:**
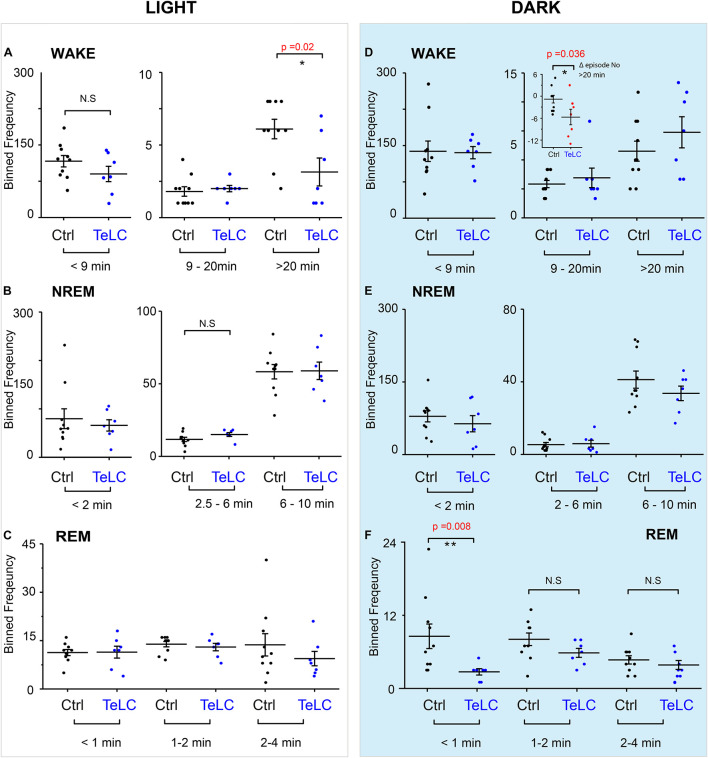
Distribution of sleep episode lengths is reduced for *Nos1-MnPO/MPO-GFP-TeLC* mice during wakefulness in the light period but increased in wakefulness in the dark period. **(A)** Wakefulness in the light period binned by episode length. No differences are seen in bins less than 20 min. Episodes greater than 20 min in *Nos1*-*MnPO/MPO-GFP-TeLC* mice (*n* = 7 TeLC and *n* = 10 GFP, *p* = 0.02). **(B)** NREM in the light period binned by episode length. No differences are seen in bins less than 10 min (*n* = 7 TeLC and *n* = 10 GFP). **(C)** Light phase REM. No differences are seen in bins less than 4 min (*n* = 7 TeLC and *n* = 10 GFP). **(D)** Dark phase wakefulness. No differences are seen between groups within each light cycle (*n* = 7 TeLC and *n* = 10 GFP). Inset graph, between the light periods *Nos1*-*MnPO/MPO-GFP* and *Nos1*-*MnPO/MPO-GFP-TeLC* groups as a paired difference (d, inset graph, *n* = 7 TeLC and *n* = 10 GFP, *p* = 0.036). **(E)** Dark phase NREM. No differences are seen in bins less than 10 min (*n* = 7 TeLC and *n* = 10 GFP). **(F)** Dark phase REM. No differences are seen in bins greater than 1 min. Episodes of less than 1 min are significant between *Nos1*-*MnPO/MPO-GFP-TeLC* and *Nos1*-*MnPO/MPO-GFP* mice (*n* = 7 TeLC and *n* = 10 GFP, *p* = 0.008). Multiple comparisons were accounted for using the Benjamini-Hochberg procedure at a false discovery rate of 5%. *P < 0.05, **P < 0.01, N.S, not significant.

Because we did not observe an overall change in the number of wake or NREM episodes during the light or dark phase, we looked at the episode length and number of vigilance states to see if this explained the differences seen in the time spent sleeping (Figure 4). In *Nos1-MnPO/MPO-GFP-TeLC* mice we observed reductions of approximately 50% in the frequency of the longest wake episodes (>20 min) in the light phase (Figure 4A), although no changes were seen in episode length and number for NREM and REM sleep (Figures 4B,C). In the dark phase, the average values for wake did not change, and the data had larger variance (Figure 4D). However, while *Nos1-MnPO/MPO-GFP* control mice had a reduced frequency of episodes, >20 min in the dark phase compared with the light phase, the *Nos1-MnPO/MPO-GFP-TeLC* mice instead had an increased frequency of these episodes. From calculating the paired difference for each mouse between the light and dark phase, it was clear that *Nos1-MnPO/MPO-GFP-TeLC* mice were more affected by the light change (Figure 4D, inset graph). No further alterations were seen in episode length and number for NREM and REM sleep during lights OFF (Figures 4E,F).

### Medial Preoptic Nitric Oxide Synthase 1 Neurons Contribute to Theta Power During REM Sleep

**FIGURE 5:**
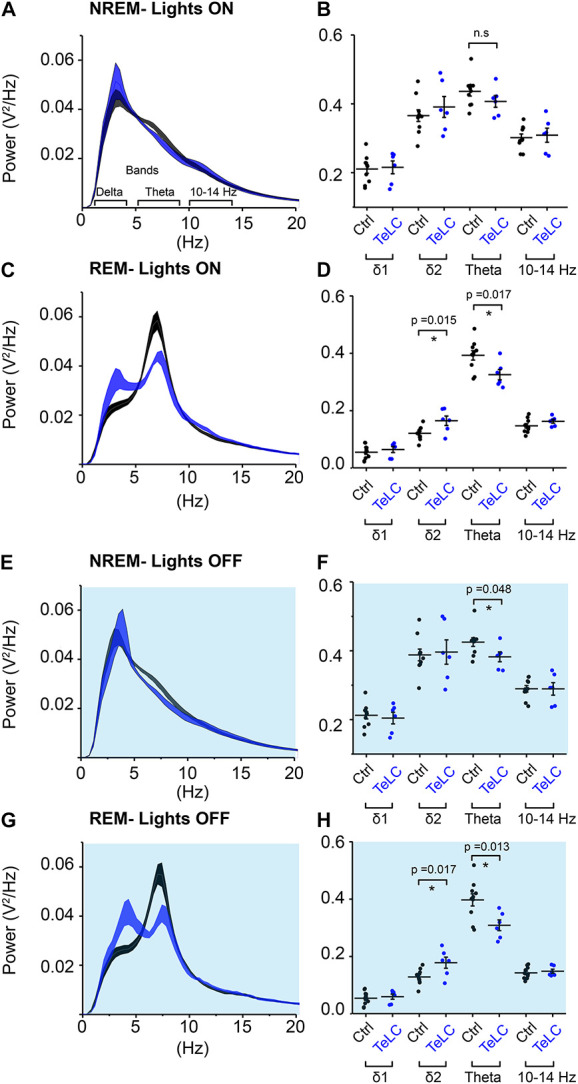
NOS1 neurons in MnPO/MPO contribute to theta power during REM sleep. **(A)** Normalized power for NREM during the light period shown as a power spectrum. Different frequency bands are also illustrated. **(B)** For NREM during the light period, data for individual mice are shown for relevant frequency bands. **(C)** REM during the light period. **(D)** Quantification of spectral differences during REM in the light period for individual mice show more δ2 in the TeLC condition (*t* test, *n* = 6, TeLC; *n* = 10, GFP; *p* = 0.015) alongside reduced theta (*t* test, *n* = 6, TeLC; *n* = 10, GFP; *p* = 0.017). **(E)** Normalized power for NREM in the dark period shown as a power spectrum. **(F)** Data for individual mice are shown for relevant frequency bands (*t* test, *n* = 6, TeLC; *n* = 10, GFP; *p* = 0.048). **(G)** REM during the dark period. **(H)** Quantification of spectral differences during REM in the light period for individual mice (δ2 between groups, *t* test, *n* = 6, TeLC; *n* = 10, GFP; *p* = 0.017). Theta between groups (*t* test, *n* = 6, TeLC; *n* = 10, GFP; *p* = 0.013). Multiple comparisons were accounted for using the Benjamini-Hochberg procedure at a false discovery rate of 5%. *P < 0.05, n.s, not significant.

We analyzed the sleep-state specific power spectra of *Nos1-MnPO/MPO-GFP-TeLC* mice compared with *Nos1-MnPO/MPO-GFP* control mice, following normalization to WAKE power within each mouse. During the lights-on phase, NREM sleep was not associated with changes in power (Figures 5A,B); however, REM sleep did show significant changes (Figure 5C). Specifically, there was an increase in delta power, normally associated with NREM sleep, of approximately 30%, as well as a corresponding decrease in theta power (Figure 5D). This difference was in the 2–4 Hz range of delta power referred to as the δ2 band (Hubbard et al., 2020). Theta (6–9 Hz) power was reduced by approximately 20% but no changes in the higher frequencies (10–14 Hz) were seen. During the dark phase (Figures 5E,F), NREM showed an approximately 15% reduction in theta power. This contrasted with no change in this band during the light phase. For REM sleep in the dark phase (Figures 5G,H), differences between *Nos1-MnPO/MPO-GFP-TeLC* and *Nos1-MnPO/MPO-GFP* mice mirrored those seen in the light phase, with an approximately 25% increase in δ2 power as well as a 20% decrease in theta power.

### Medial Preoptic Nitric Oxide Synthase 1 Neurons Reduce Body Temperature

**FIGURE 6:**
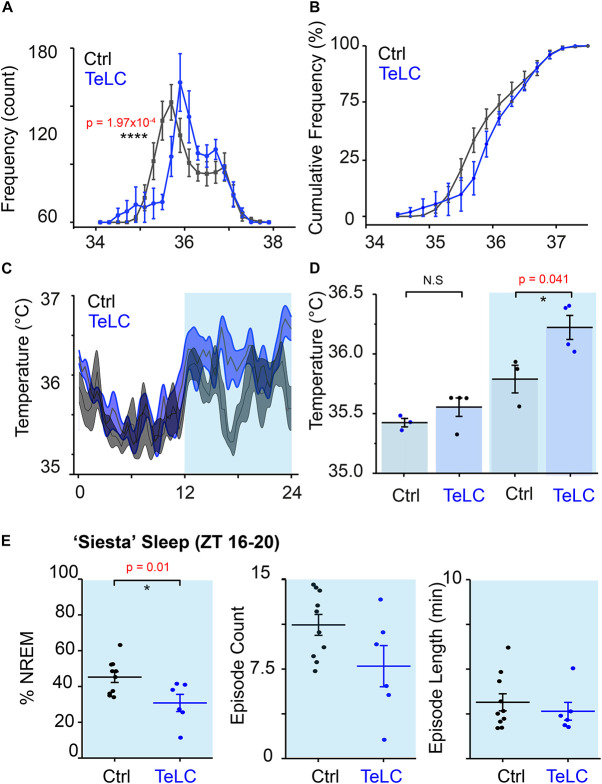
NOS1 neurons in MnPO/MPO hypothalamus act to reduce body temperature. **(A)** Aggregated temperature data from 7 days of recording for each mouse averaged by ZT time at 2-min intervals to produce a ‘typical’ day histogram, averaged for each group (rmANOVA, *n* = 4, TeLC; *n* = 6, GFP; temperature × group at 35.4–35.6°C, *p* = 1.97 × 10^–4^). **(B)** Cumulative frequency of temperature distribution. **(C)** Temperature profile over 24 h. **(D)** Quantification of 24-h temperature profile in the light and dark phase (two-tailed *t* test assuming unequal variance, *n* = 4, TeLC; *n* = 3, GFP; *p* = 0.041). **(E)** Percentage sleep and episode count and episode length a ‘siesta’ period between ZT 16–20 (two tailed *t*-test, *n* = 7 and *n* = 10, *p* = 0.01). *P < 0.05, N.S, not significant.

Using implanted temperature loggers, we measured the core body temperature at 2-min resolution in *Nos1-MnPO/MPO-GFP-TeLC* and *Nos1-MnPO/MPO-GFP* mice (Figure 6). We produced a typical 24-h period in temperature change by first averaging over 7 days for each mouse (5040 measurements) before comparing distributions across groups. The temperature distribution of *Nos1-MnPO/MPO-GFP-TeLC* mice shifted to warmer temperatures compared with those of control mice (Figure 6A). The cumulative distribution illustrated that the most significant change was in the probability of observing core temperature between 35.5 and 36°C, but without a change in the minimum or maximum temperatures (Figure 6B). Furthermore, during the dark phase, while *Nos1-MnPO/MPO-GFP* control mice dropped their core temperature, *Nos1-MnPO/MPO-GFP-TeLC* control mice did not, both during the middle of the dark phase and prior to the next lights-on period (Figures 6C,D); furthermore, during the siesta period *Nos1-MnPO/MPO-GFP-TeLC* mice spent 36% less total time in NREM sleep compared with control mice, although there were no significant changes in NREM sleep episode count or mean length (Figure 6E).

## Discussion

In this study we have discovered that many NOS1 cells in the midline PO hypothalamus are naturally sleep-active, although there seemed to be several different categories of responses and probably several different types of cell. Based on their calcium signals, the fast and transient “sharp” activations of these NOS1 cells from wake to NREM and the slower, prolonged “soft” transitions from wake to NREM may represent two populations of sleep-active NOS1 neurons. However, the cells are not active throughout NREM sleep, but instead are most active at the transitions from wake to NREM and the subsequent first part of NREM. They also become active during the later parts of REM sleep. The “sharp transition” subgroup is more likely to play a role in sleep onset, whereas the other groups appear to be following with their activity after NREM and REM sleep are established. The cells are not silent during wake periods, but their activity is intermittent. A minority (10%) of these NOS1 cells show no change in their calcium signals at the transitions of the vigilance states, again suggesting subtypes of cells. We used TeLC expression in NOS1 neurons to disrupt their synaptic activity, which in turn disrupted the sleep-wake profile of the mice in a manner that varied with the light or dark phases of the 24-h cycle. In the dark phase, mice with TeLC expressed in MnPO/MPO NOS1 neurons showed a reduction of time in NREM sleep and a loss of the shortest REM episodes; NREM to REM and REM to wake transitions were also reduced. REM sleep was accompanied by increased delta power and decreased theta power, possibly suggesting functional disruption of REM sleep. In the lights-on phase, however, there was an increase in NREM sleep, but REM sleep was unchanged. Overall, the mice were chronically warmer.

Our new results are consistent with our previous work on these cells. We have previously shown that a subset of MnPO/MPO glutamate/NOS1 neurons, when activity-tagged following an external warm-stimulus to the mice, could on reactivation induce NREM sleep and concomitant body cooling (Harding et al., 2018). Similarly, a GABAergic MnPO/MPO population, tagged in the same manner, could only induce sleep (Harding et al., 2018). As we did not observe overlap in these populations by immunohistochemistry, we suggested a model of external warmth-triggered sleep with a NOS1/glutamate (MnPO/MPO) population signaling to a downstream GABAergic population in MPO (Harding et al., 2020). Thus MnPO/MPO NOS1 neurons can sense changes in temperature although we do not know if this is direct sensing or through afferents from the skin. In *Nos1-MnPO/MPO-GFP-TeLC* mice, contrary to the expectation that NREM sleep would be unchanged or reduced during the light phase, these mice had an increase in the light phase, and a subsequent reduction in this state during the dark phase. So, it is possible that these changes in sleep-wake states result from the altered thermoregulation, or the effect is complex because of the likely multiple subtypes of cell. The effects on REM sleep (selective for the dark phase) were unanticipated, but perhaps not surprising given that REM sleep is partly controlled by unknown cell types in the MPO area (Suntsova and Dergacheva, 2004; Gvilia et al., 2006), and we have presumably influenced a NOS1 cell subtype involved in REM production. Alternatively, the reduction in REM sleep when NOS1 neurons are blocked might be linked to the reduction of NREM sleep, which both happen in the dark phase, and not as a result of a REM-specific mechanism. This idea is consistent with a lower theta power during REM, which may indicate reduced REM sleep propensity.

Initially, given that *nos1* gene expression in the MPO hypothalamus has a highly restricted expression pattern, as detected by both *in situ* hybridization and immunohistochemistry (see e.g., Figure 4E in Harding et al., 2018), we anticipated that *nos1* expression would be a pragmatic and useful marker for functional manipulation of a unique subset of cells. Unfortunately, this has turned out not to be the case. While many of the NOS1 neurons in MPO studied by calcium photometry have clear sleep-active patterns, it has become apparent since we started our work that multiple subtypes of NOS1 neuron exist in the PO area, including NOS1/VGLUT2, NOS1/VGAT, NOS1/galanin neurons and others (Moffitt et al., 2018). The bidirectional changes in sleep when TeLC is expressed in MnPO/MPO NOS1 neurons likely reflect the reduced synaptic transmitter release from multiple subtypes of NOS1 cell in MnPO/MPO. For example, activation of glutamate (VGLUT2) neurons in the PO area induces wakefulness (Vanini et al., 2020), so if this particular subset were to express the *nos1* gene, TeLC expression in them might reduce wakefulness; on the other hand, we have shown previously that *nos1*-expressing GABA cells induce NREM sleep (Harding et al., 2018); thus TeLC expression in NOS1 cells might promote wakefulness (Harding et al., 2018). Further progress to dissect this circuitry requires intersectional genetics. Nevertheless, it remains striking that the majority of MnPO/MPO NOS1 cells have most of their activity during the transitions from wake to NREM sleep, and during the later parts of REM sleep episodes. Targets for NOS1 neurons could include GABAergic and galaninergic neurons in the LPO area that are involved in NREM sleep induction and maintenance (Kroeger et al., 2018; Ma et al., 2019), as well as uncharacterized long-range targets. Although we always used the same coordinates, we did not attempt to distinguish NOS1 cells in the small and neighboring MnPO and MPO areas.

Expressing TeLC in MnPO/MPO NOS1 neurons raised the average body temperature of the mice. This would be consistent with effects on temperature mediated by BDNF/PACAP or TRPM2 expressing neurons in the MPO area (Song et al., 2016; Tan et al., 2016; Harding et al., 2018); these neurons could co-express NOS1. There are also glutamatergic wake-promoting neurons in the PO that are associated with mild body cooling of approximately 1°C that could also have a role (Vanini et al., 2020), and may also express the *nos1* gene. However, unlike the effects on temperature produced by BDNF/PACAP or TRPM2 cells, the increases we see appear to be associated with the light phase of the dark-light cycle, specifically in the siesta period (ZT16-20) and the period before the dark-to-light transition. Thus, MnPO/MPO NOS1 cells are driving down temperature at the same time as NREM sleep is initiated, consistent with our earlier work (Harding et al., 2018). Overall, this may support a larger hypothesis on optimization of sleep for energy reallocation (Harding et al., 2020).

In summary, we have found that the activity pattern of some MnPO/MPO NOS1 cells is quite striking, being rather selective at the boundary between wake to NREM transitions and the later part of REM sleep, and that synaptic transmission from PO NOS1 neurons likely contributes to NREM and REM sleep organization, as well as chronic body cooling. We write “likely” because we have not formally shown that TeLC expression reduced transmitter release in these neurons, and we have not identified post-synaptic targets of MnPO/MPO NOS1 cells. We currently think that MnPO/MPO NOS1 neurons probably have both short local outputs and long-range connections where transmitters could be released. A further caveat is that NO itself is likely to be part of the signaling system from these cells. We did not address this because NO release from cells is independent of vesicle release. But as NOS1 synthase is calcium-dependent (Knowles and Moncada, 1994), periods of elevated calcium seen in NOS1 neurons at the wake to NREM transitions and during NREM sleep will result in NO release from these cells, and NO could well be influencing sleep structure and temperature regulation. We further speculate that NOS1 neurons, possibly using NO release, may have a role in controlling vasodilation specifically in the context of sleep. We should bear in mind that we have only looked at male mice, and because the PO area is sexually dimorphic, NOS neurons could differ in their effects between the sexes. Given the rather precise calcium activity of some MnPO/MPO NOS1 cells at the boundary of wake to NREM transitions, further dissection will likely reveal part of a regulatory circuit controlling sleep induction/maintenance and the simultaneous lowering of body temperature.

## Data Availability Statement

The raw data supporting the conclusions of this article will be made available by the authors, without undue reservation.

## Ethics Statement

The animal study was reviewed and approved by Imperial College AWERB Committee and United Kingdom Home Office.

## Author Contributions

WW, NF, and EH conceived the project. EH with input from WW and NF designed the experiments. EH, WB, RZ, XY, RY, and LL performed the experiments. RZ and LL were supervised by EH for this work. BH and TC provided technology for EEG recordings. FM helped with the manuscript and data analysis. AV provided the Neurologgers. EH performed the data analysis and produced the figures. NF and WW contributed to the data analysis and supervised the project. EH with NF and WW wrote the manuscript. All authors contributed to the article and approved the submitted version.

## Conflict of Interest

The authors declare that the research was conducted in the absence of any commercial or financial relationships that could be construed as a potential conflict of interest.

## Publisher’s Note

All claims expressed in this article are solely those of the authors and do not necessarily represent those of their affiliated organizations, or those of the publisher, the editors and the reviewers. Any product that may be evaluated in this article, or claim that may be made by its manufacturer, is not guaranteed or endorsed by the publisher.
